# Supplementary material for: Leveraging the histidine kinase-phosphatase duality to sculpt two-component signaling
Source: Nat Commun. 2024 Jun 10;15:4876. doi: 10.1038/s41467-024-49251-8 (PMC11164954; doi:10.1038/s41467-024-49251-8)
Supplement: Supplementary file 1 — Supplementary Information [file 41467_2024_49251_MOESM1_ESM.pdf]

Leveraging the Histidine Kinase-Phosphatase Duality to Sculpt Two-Component Signaling

Stefanie S. M. Meier[1,†,‡], Elina Multamäki[2,†,‡], Américo T. Ranzani[1,‡], Heikki Takala[2,3,*,‡], Andreas Möglich[1,4,5,*,‡]

[1] Department of Biochemistry, University of Bayreuth, 95447 Bayreuth, Germany.
[2] Department of Anatomy, University of Helsinki, Helsinki 00014, Finland.
[3] Department of Biological and Environmental Science, Nanoscience Center, University of Jyvaskyla, Jyvaskyla 40014, Finland.
[4] Bayreuth Center for Biochemistry & Molecular Biology, Universität Bayreuth, 95447 Bayreuth, Germany.
[5] North-Bavarian NMR Center, Universität Bayreuth, 95447 Bayreuth, Germany.

[†] These authors contributed equally to this work.
* for correspondence: heikki.p.takala@jyu.fi, andreas.moeglich@uni-bayreuth.de
[‡] ORCID identifiers: S.M. 0009-0002-6028-5223; E.M. 0000-0002-0636-9269; A.T.R. 0000-0002-6203-9467; H.T. 0000-0003-2518-8583; A.M. 0000-0002-7382-2772

Table of Contents

Supplementary Table 1 – Plasmid Systems for Red-Light-Controlled Bacterial Gene Expression ___ 3

Supplementary Table 2 – Oligonucleotide Primers Used in the Study ________________________ 4

Supplementary Figure 1 ____ 9

Supplementary Figure 2 ____ 10

Supplementary Figure 3 ____ 11

Supplementary Figure 4 ____ 12

Supplementary Figure 5 ____ 13

Supplementary Figure 6 ____ 14

Supplementary Figure 7 ____ 15

Supplementary Figure 8 ____ 17

Supplementary Figure 9 ____ 18

Supplementary Figure 10 ____ 19

Supplementary Figure 11 ____ 20

Supplementary References ____ 21

Supplementary Table 1 – Plasmid Systems for Red-Light-Controlled Bacterial Gene Expression

Construct	PSM	DHp/CA	Activated by	Addgene ID	Reference
*Dm*REDusk	*Dm*PSM (1-510)	FixL (266-505)	darkness	213701	this work
*Dr*REDusk	*Dr*PSM (1-506)	FixL (266-505)	darkness	188970	ref. [1]
*Dm*REDawn	*Dm*PSM (1-510)	FixL (266-505)	red light	n/a	this work
*Dr*REDawn	*Dr*PSM (1-506)	FixL (266-505)	red light	188971	ref. [1]
*Dm*DERusk	*Dm*PSM (1-510)	FixL (284-505)	red light	213702	this work
*Dr*DERusk	*Dr*PSM (1-505)	FixL (271-505)	red light	213703	this work

Supplementary Table 2 – Oligonucleotide Primers Used in the Study

Name	Sequence
primers for Gibson cloning of *Dm*PSM into *Dr*REDusk and *Dr*REDawn	
QX_DmDuskVec_F	AAGTTCAGGGTGCCCGTCTGCTCCAGGAACTGCAATCCGA
QX_DmDuskVec_R	ACGCGCTGCCGGACGTTCTGTCATGCGTGGGCGACCTCAG
QX_DmPCM_F	CTGAGGTCGCCCACGCATGACAGAACGTCCGGCAGCGCGT
QX_DmPCM_R	TCGGATTGCAGTTCCTGGAGCAGACGGGCACCCTGAACTT
primers for cloning PATCHY start constructs	
A7_P_I_II_Step1_for	TTAACCGAGCATCAGCAGACACAAGCACGTCTCCAGGAACTGCAATCCGAGCTCG
A11_P_I_Step1_rev	GATTCAGATCCCGGATGACCGACAGGCGCTCGCCCAGCGCCC
A78_P_I_Step2_for	CGAGTGGCGGCAGTACGGGTTTGTTAACCGAGCATCAGCAGACACAAG
A79_P_I_Step2_rev	GCGTTAGATTGAGTCAGCGCACGATTCAGATCCCGGATGACCGAC
A13_P_II_Step1_rev	TAAGCGTGCATTGAGGTCCTGCAACGCGGCCAGACGGGCACCCTGAACTTCCA
A76_P_II_Step2_for	TAACGCAGAGTTGCGGCGTTTCGCCTTTGTTAACCGAGCATCAGCAGACACAAG
A77_P_II_Step2_rev	CGCCCTTCAAGCTCACGATTAGCACGCTCTAAGCGTGCATTGAGGTCCTGC
PATCHY forward primers for amplification of *Bj*FixL	
A14_P_I_II_for_1	ACCGAGCATCAGCAGAC
A15_P_I_II_for_2	GAGCATCAGCAGACACAAG
A16_P_I_II_for_3	CATCAGCAGACACAAGC
A17_P_I_II_for_4	CAGCAGACACAAGCACG
A18_P_I_II_for_5	CAGACACAAGCACGTCTC
A19_P_I_II_for_6	ACACAAGCACGTCTCC
A20_P_I_II_for_7	CAAGCACGTCTCCAGG
A21_P_I_II_for_8	GCACGTCTCCAGGAAC
A22_P_I_II_for_9	CGTCTCCAGGAACTGC
A23_P_I_II_for_10	CTCCAGGAACTGCAATCC
A24_P_I_II_for_11	CAGGAACTGCAATCCG
A25_P_I_II_for_12	GAACTGCAATCCGAGC

Name	Sequence
A26_P_I_II_for_13	CTGCAATCCGAGCTCG
A27_P_I_II_for_14	CAATCCGAGCTCGTCC
A28_P_I_II_for_15	TCCGAGCTCGTCCACG
A29_P_I_II_for_16	GAGCTCGTCCACGTCTC
A30_P_I_II_for_17	CTCGTCCACGTCTCCAG
A31_P_I_II_for_18	GTCCACGTCTCCAGGC
A32_P_I_II_for_19	CACGTCTCCAGGCTGAG
A33_P_I_II_for_20	GTCTCCAGGCTGAGCG
A34_P_I_II_for_21	TCCAGGCTGAGCGCCATG
A35_P_I_II_for_22	AGGCTGAGCGCCATGG
A36_P_I_II_for_23	CTGAGCGCCATGGGCG
A37_P_I_II_for_24	AGCGCCATGGGCGAAATG
A38_P_I_II_for_25	GCCATGGGCGAAATGG
A39_P_I_II_for_26	ATGGGCGAAATGGCGTC
A40_P_I_II_for_27	GGCGAAATGGCGTCCG
A41_P_I_II_for_28	GAAATGGCGTCCGCGC
A42_P_I_II_for_29	ATGGCGTCCGCGCTCG
PATCHY reverse primers for amplification of *Dm*PSM	
A43_P_II_rev_1	AAAGGCGAAACGCCGC
A44_P_II_rev_2	GGCGAAACGCCGCAAC
A45_P_II_rev_3	GAAACGCCGCAACTCTG
A46_P_II_rev_4	ACGCCGCAACTCTGCG
A47_P_II_rev_5	CCGCAACTCTGCGTTAC
A48_P_II_rev_6	CAACTCTGCGTTACGC
A49_P_II_rev_7	CTCTGCGTTACGCCCTTC
A50_P_II_rev_8	TGCGTTACGCCCTTCAAG
A51_P_II_rev_9	GTTACGCCCTTCAAGC
A52_P_II_rev_10	ACGCCCTTCAAGCTCAC
A53_P_II_rev_11	CCCTTCAAGCTCACGATTAG
A54_P_II_rev_12	TTCAAGCTCACGATTAGC

Name	Sequence
A55_P_II_rev_13	AAGCTCACGATTAGCACG
A56_P_II_rev_14	CTCACGATTAGCACGC
A57_P_II_rev_15	ACGATTAGCACGCTCTAAGC
A58_P_II_rev_16	ATTAGCACGCTCTAAGCG
A59_P_II_rev_17	AGCACGCTCTAAGCGTG
A60_P_II_rev_18	ACGCTCTAAGCGTGCATTG
A61_P_II_rev_19	CTCTAAGCGTGCATTGAGG
A62_P_II_rev_20	TAAGCGTGCATTGAGG
A63_P_II_rev_21	GCGTGCATTGAGGTCC
A64_P_II_rev_22	TGCATTGAGGTCCTGC
A65_P_II_rev_23	ATTGAGGTCCTGCAACG
A66_P_II_rev_24	GAGGTCCTGCAACGCG
A67_P_II_rev_25	GTCCTGCAACGCGGCC
A68_P_II_rev_26	CTGCAACGCGGCCAGAC
A69_P_II_rev_27	CAACGCGGCCAGACGG
A70_P_II_rev_28	CGCGGCCAGACGGGCAC
A71_P_II_rev_29	GGCCAGACGGGCACCC
A72_P_II_rev_30	CAGACGGGCACCCTGAAC
A73_P_II_rev_31	ACGGGCACCCTGAACTTC
A74_P_II_rev_32	GGCACCCTGAACTTCC
A75_P_II_rev_33	ACCCTGAACTTCCAGTAATGC
PATCHY reverse primers for amplification of *Dr*PSM	
flib1_3766_rev	AAACCCGTACTGCCGC
flib1_3763_rev	CCCGTACTGCCGCCAC
flib1_3760_rev	GTACTGCCGCCACTCG
flib1_3757_rev	CTGCCGCCACTCGGCG
flib1_3754_rev	CCGCCACTCGGCGTTAG
flib1_3751_rev	CCACTCGGCGTTAGATTG
flib1_3748_rev	CTCGGCGTTAGATTGAGTC
flib1_3745_rev	GGCGTTAGATTGAGTCAGC

Name	Sequence
flib1_3742_rev	GTTAGATTGAGTCAGCGCAC
flib1_3739_rev	AGATTGAGTCAGCGCACG
flib1_3736_rev	TTGAGTCAGCGCACGATTC
flib1_3733_rev	AGTCAGCGCACGATTCAG
flib1_3730_rev	CAGCGCACGATTCAGATC
flib1_3727_rev	CGCACGATTCAGATCCC
flib1_3724_rev	ACGATTCAGATCCCGG
flib1_3721_rev	ATTCAGATCCCGGATGACC
flib1_3718_rev	CAGATCCCGGATGACC
flib1_3715_rev	ATCCCGGATGACCGAC
flib1_3712_rev	CCGGATGACCGACAGG
flib1_3709_rev	GATGACCGACAGGCGC
flib1_3706_rev	GACCGACAGGCGCTCG
flib1_3703_rev	CGACAGGCGCTCGCCC
flib1_3700_rev	CAGGCGCTCGCCCAGC
flib1_3697_rev	GCGCTCGCCCAGCGCC
flib1_3694_rev	CTCGCCCAGCGCCCCG
flib1_3691_rev	GCCCAGCGCCCCGGTC
primers for *Dm*DERusk-YPet	
AGF_1_BB_to_Ypet_rev	GGTGGTGGTGCTCGAGTGCGGCCGCAAGCTTCTATTTATACAGTTCATTCATACCTTCGG
AGF_2_Ypet_to_Bb_rev	CCGAAGGTATGAATGAACTGTATAAATAGAAGCTTGCGGCCGCACTCGAGCACCACCACC
AGF_3_BB_to_Ypet_for	CAGCAGCGGCCTGGTGCCGCGCGGCAGCCATATGGTTAGCAAAGGTGAAGAACTGTTTAC
AGF_4_Ypet_to_BB_for	GTAAACAGTTCTTCACCTTTGCTAACCATATGGCTGCCGCGCGGCACCAGGCCGCTGCTG
primers for cloning of *Dm*REDusk-MCS	
A97_Ins_for	GTGCCTGAGGTCGCCCACGCATGACAGAACGTCCGGCAGCG
A98_Ins_rev	GCTCGGATTGCAGTTCCTGGAGCAGACGGGCACCCTGAACTTCCAGT

Name	Sequence
A99_Bb_for	ACTGGAAGTTCAGGGTGCCCGTCTGCTCCAGGAACTGCAATCCGAGC
B01_Bb_rev	CGCTGCCGGACGTTCTGTCATGCGTGGGCGACCTCAGGCAC
primers for cloning of *Dm*DERusk-MCS	
A97_Ins_for	GTGCCTGAGGTCGCCCACGCATGACAGAACGTCCGGCAGCG
B01_Bb_rev	CGCTGCCGGACGTTCTGTCATGCGTGGGCGACCTCAGGCAC
B26_Ins_rev	CGAGATGATAGGAGGTCTAGCATGACGACCAAGGGACATATCTACG
B27_Bb_for	CGTAGATATGTCCCTTGGTCGTCATGCTAGACCTCCTATCATCTCG
primers for cloning of pCDF expression constructs for *Dm*F1 and *Dm*F1+23	
B12_EC_DmF1_Bb_for	GCCGACGAGATGATAGGAGGTCTAGCACATCACCATCACCATCACCATCAC
B13_EC_DmF1_Bb_rev	GCGCTGCCGGACGTTCTGTCATGGTATATCTCCTTATTAAAGTTAAACAAA
B14_EC_DmF1_Ins_for	TTTGTTTAACTTTAATAAGGAGATATACCATGACAGAACGTCCGGCAGCGC
B15_EC_DmF1_Ins_rev	GTGATGGTGATGGTGATGGTGATGTGCTAGACCTCCTATCATCTCGTCGGC
sequencing primers	
A5_DdDusk_Seq_for	AAGTGCTGGAAGCCAGCGTG
A6_DdDusk_Seq_rev	ATGTAGTTGCTGATCGCCGC
A80_P_I_II_Seq	GTGTGTTCGGATCACTGC
A89_Seq_ACYCDuetUP1	GGATCTCGACGCTCTCCCT
B28_Seq_in_FixJ	AGGGCGTCGAGAAAGG

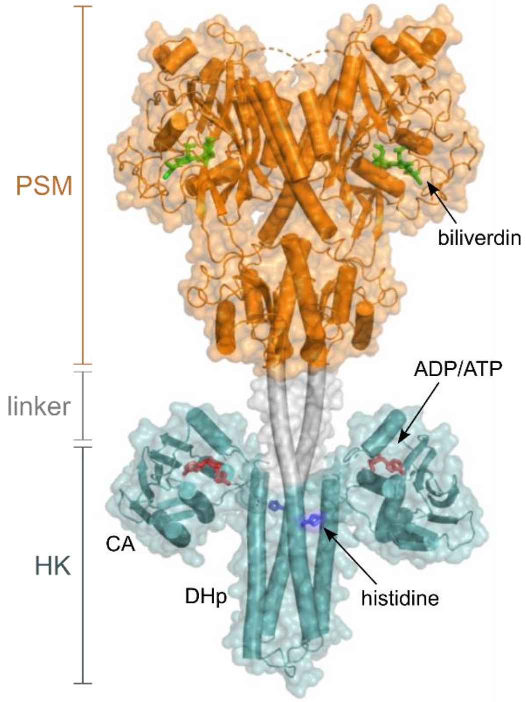

Supplementary Figure 1

Structural model of the bacteriophytochrome from *D. radiodurans* (*Dr*BphP), a paradigm red-light-sensing SHK. *Dr*BphP consists of a biliverdin-binding photosensory module (PSM), which is connected through a coiled-coil linker to an output histidine kinase (HK) module. The HK in turn consists of DHp (dimerization and histidine phosphotransfer) and CA (catalytic and ATP binding) subdomains. The active-site histidine and bound adenosine nucleotides are indicated. The model is derived from the cryo-EM structure of *Dr*BphP (PDB ID: 8AVW [http://doi.org/10.2210/pdb8AVW/pdb]) [2] and the crystal structure of the effector moiety of *Thermotoga maritima* HK853 (2C2A [http://doi.org/10.2210/pdb2C2A/pdb]) [3].

```
DrPSM      MSRDPLPFFPPLYLGGPEITTENCEREPIHIPGSIQPHGALLTADGHSGEVLQMSLNAAT 60
DmPSM      -MTGGDDFLTPAHLGGPPVDTDNCAREPIHIPGAVQPHGALLVLRADDERAVQVSANSEA 59
              .   *: * :**** : *:** ********::*******.  ... ..:*:* *: :
                                                                          PAS

DrPSM      FLGQEPTVLRGQTLAALLPEQWPA-LQAALPPGCPDALQYRATLDWPAAGHLSLTVHRVG 119
DmPSM      FVGVAPDALLGQHLADLVGDQGAAALRAAFAQAGPS----AMTLTFRNGRTYDVTARRDG 115
           *:*  * .* ** ** *: :*  * *:**:  . *.      ** :  .   .:*.:* *

DrPSM      ELLILEFEPTEAWDST--GPHALRNAMFALESAPNLRALAEVATQTVRELTGFDRVMLYK 177
DmPSM      ELLLMELEPPDVRAGTPALYHAIRDALGALEHAPDLHALLDVAAQRVRALTGLDRVMIYR 175
           ***::*:** :.  .*    **:*:*: *** **:*:** :**:* ** ***:****:*:

DrPSM      FAPDATGEVIAEARREGLHAFLGHRFPASDIPAQARALYTRHLLRLTADTRAAAVPLDPV 237
DmPSM      FAADDSGEVVAEARAPHLHAFLGHRFPESDIPRQARALYVQHLLRFTADAGGGQVPLVPA 235
           ** * :***:****   ********** **** ******.:****:***: .. *** *.
                                                                          GAF

DrPSM      LNPQTNAPTPLGGAVLRATSPMHMQYLRNMGVGSSLSVSVVVGGQLWGLIACHHQTPYVL 297
DmPSM      LNPVTNAPLQMGALVLRATSPVHLQYLRNMGVIASMSVSIVQDGRLWGLIACHHGAAHVV 295
           *** ****  :*. *******:*:******** :*:***:* .*:********* : :*:

DrPSM      PPDLRTTLEYLGRLLSLQVQVKEAADVAAFRQSLREHHARVALAAAHSLSPHDTLSDPAL 357
DmPSM      PQATRDACEFLGRVLSLQITAKRDAAVNARRAALGAQHARLVTAVTGTLTPLDALTRADL 355
           *   * : *:***:****: .*. * * * * :*  :***:. *.: :*:* *:*:   *

DrPSM      DLLGLMRAGGLILRFEGRWQTLGEVPPAPAVDALLAWLETQPGALVQTDALGQLWPAGAD 417
DmPSM      NLPGFLDTAGAAVRMDGGTRTLGVTPSDEDLEHLVAWLRAQGTPSVCTDALARTYPPGAL 415
           :* *:: :.*  :*::*  :*** .*    :: *:***.:*    * ****.: :* **
                                                                          PHY

DrPSM      LAPSAAGLLAISVGEGWSECLVWLRPELRLEVAWGGATP------DQAKDDLGPRHSFDT 471
DmPSM      FMERASGVLGVSISGNWDEYLLWFRPEIPATITWGGDPHKAVQVSDDGTARLTPRASFSS 475
           :   *:*:*.:*:. .*.* *:*:***:   ::***         *:..  * ** **.:

DrPSM      YLEEKRGYAEPWHPGEIEEAQDLRDTLTGALGERL  506
DmPSM      YVEAVRGVAQPWQAGDLDAALDLRGALLEVQGARL  510
           *:*  ** *:**: *::: * ***.:*  . * **
```

Supplementary Figure 2

Sequence alignment of the *Dm*PSM (E8U3T3 [https://www.uniprot.org/uniprotkb/E8U3T3]) and the *Dr*PSM (Q9RZA4 [https://www.uniprot.org/uniprotkb/Q9RZA4]), generated with Clustal Omega [4]. The PAS (orange, PF08446), GAF (cyan, PF01590), and PHY (green, PF00360) domains are highlighted according to the Pfam classification [5]. Red symbols show the start and end of the PHY tongue, and the characteristic PRXSF motif conserved in BphPs is highlighted in blue.

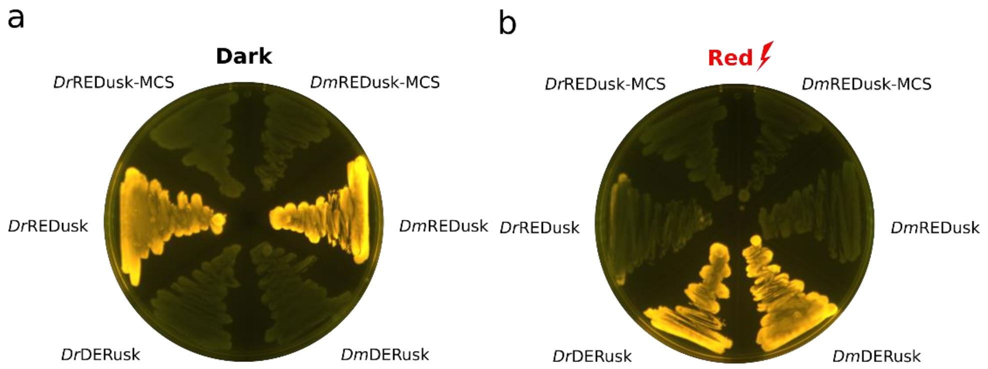

Supplementary Figure 3

Bacteria harboring the *Dm*REDusk, *Dm*DERusk, *Dr*REDusk, and *Dr*DERusk plasmids with either a *Ds*Red fluorescent reporter or a multiple-cloning site included (i.e., empty vector) were cultivated in darkness (panel **a**) or under red light (650 nm, 100 µW cm^{-2}, panel **b**). *Ds*Red production was visualized under blue light through a 520-nm long-pass filter.

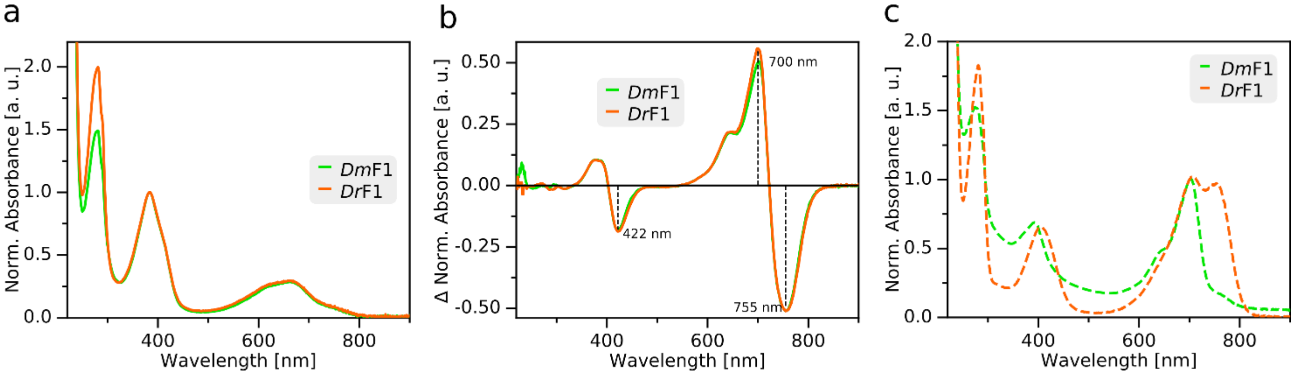

Supplementary Figure 4

Spectroscopic characterization of *Dm*F1 (green) and *Dr*F1 (orange). **a** Spectra were recorded after denaturing the samples in 6 M guanidinium chloride. The spectra were normalized to the Soret band maximum at 384 nm. **b** Difference spectra of the native proteins were calculated by subtracting the spectra upon far-red illumination from those following red-light exposure. The maxima of the Soret band (422 nm) and the Q band (700 and 755 nm) are labeled. **c** Spectra of *Dm*F1 and *Dr*F1 following illumination with saturating red light and recovery in darkness at 25°C for 24 h. Spectra were normalized to the absorbance at 700 nm. Source data are provided as a Source Data file.

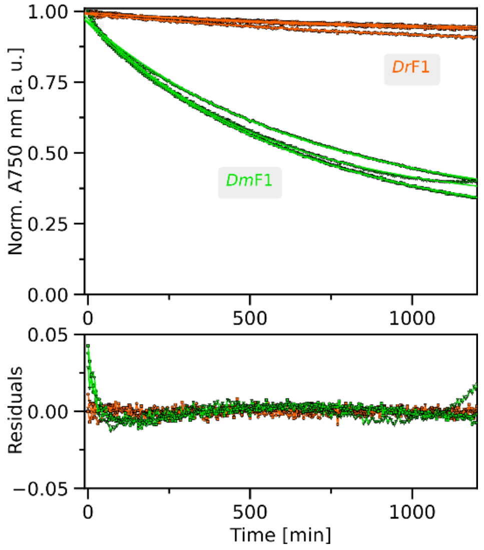

Supplementary Figure 5

Recovery kinetics of *Dm*F1 (green) and *Dr*F1 (orange) in triplicates (n = 3). Data were normalized to the maximum absorbance value and evaluated according to single-exponential functions. Source data are provided as a Source Data file.

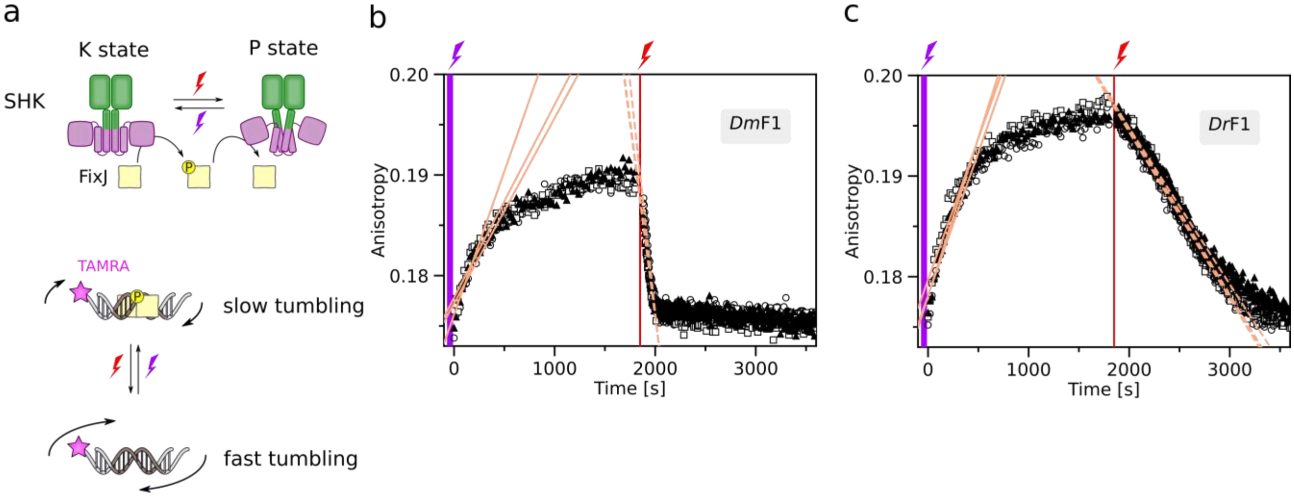

Supplementary Figure 6

Histidine kinase and phosphatase activity of *Dm*F1 and *Dr*F1 as monitored by fluorescence anisotropy. **a** Schematic of the assay. Red and far-red light interconvert *Dm*F1 and *Dr*F1 between their Pr and Pfr states which, respectively, possess net kinase (K state) and net phosphatase activity (P state) towards the response regulator FixJ. Once phosphorylated, FixJ binds to its cognate operator sequence embedded within a double-stranded DNA fragment labeled with the TAMRA fluorophore. Phospho-FixJ binding decelerates the rotational tumbling of said DNA fragment and thus incurs an increase in TAMRA fluorescence anisotropy. **b** Experimental results for *Dm*F1 in triplicates (n = 3, white circles, white squares, black triangles). See Fig. 2f for details. **c** As panel b but for *Dr*F1. Source data are provided as a Source Data file.

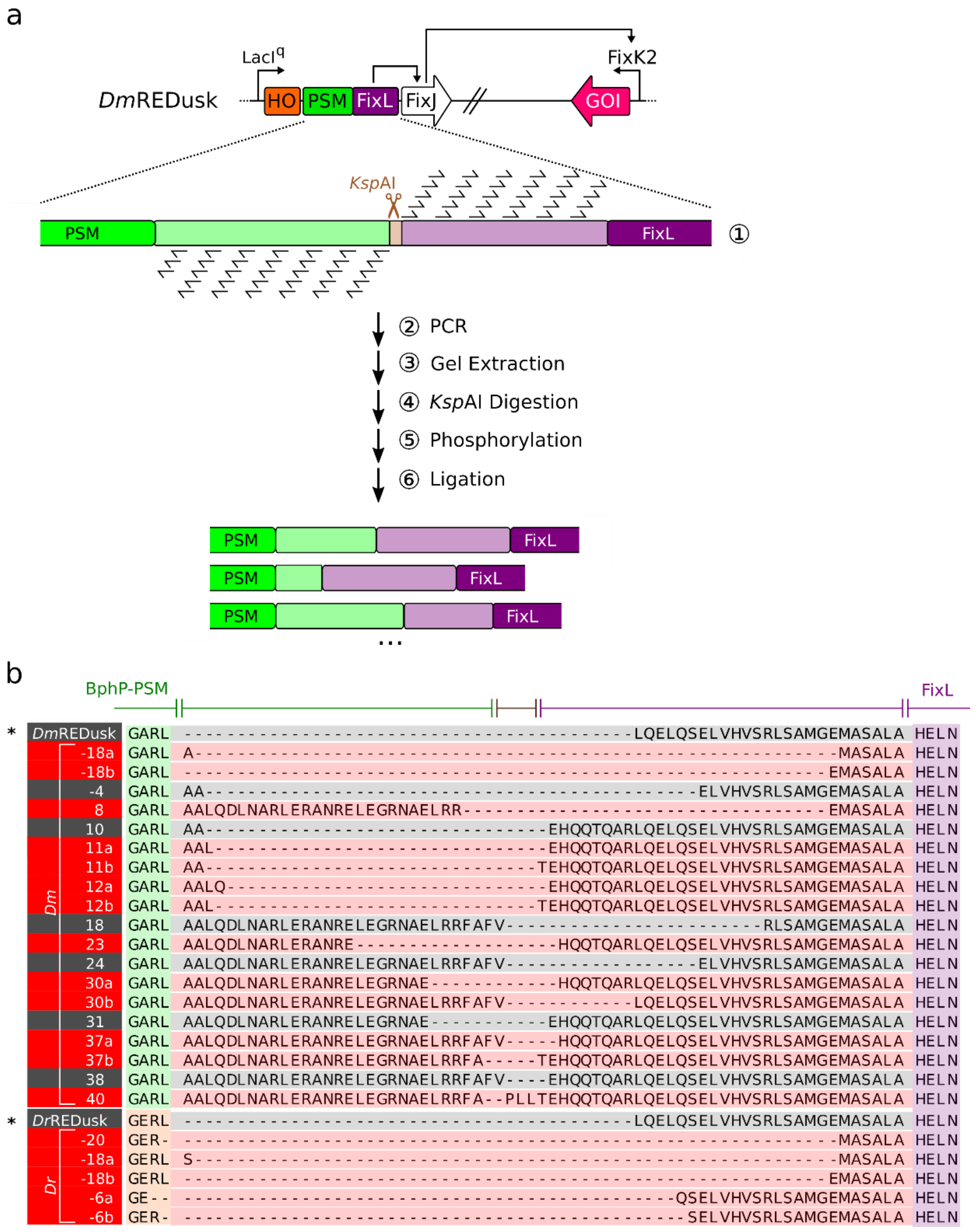

Supplementary Figure 7

Generation of derivative circuits for red-light-responsive bacterial gene expression by the PATCHY method (primer-aided truncation for the creation of hybrid proteins) [6]. **a** As exemplified for *Dm*REDusk, PATCHY resorts to a starting construct that comprises extended linker segments stemming from the parental *Deinococcus maricopensis* photosensory module (PSM, green) and the *Bradyrhizobium japonicum* FixL sensor histidine kinase (SHK, purple) (point ① in the figure). A unique restriction site (*Ksp*AI, brown) is introduced at the junction between the PSM and SHK linker

fragments. With this construct as the template, PCR amplification with sets of staggered forward and reverse primers generates a combinatorial library of linear DNA molecules that encode varying portions of the linkers of the parental PSM and SHK (②). Following DNA purification (③), the original PCR template is depleted by restriction digest (④). Phosphorylation by polynucleotide kinase (⑤) and blunt-end ligation (⑥) yield a library of circular plasmids that encode different receptor variants. Fluorescence-based screening on agar plate (see Suppl. Fig. 3) allows the identification of clones supporting light-dependent gene expression. *Dr*REDusk variants were generated likewise but rely on the *Deinococcus radiodurans* PSM rather than that of *D. maricopensis*. **b** Linker sequences of *Dm*REDusk and *Dr*REDusk variants created by PATCHY. The parental *Dm*REDusk and *Dr*REDusk constructs are indicated with an asterisk (*), and their relative linker lengths were arbitrarily assigned a value of 0. Variants with the same linker length but different sequence are designated with the suffixes ‘a’ and ‘b’. The linker length refers to the region between the indicated positions. The left part of the linker is derived from the parental *Dm*BphP/*Dr*BphP and the right part from the parental FixL histidine kinase. As indicated on the left side, several variants exhibit gene expression in darkness as the parental constructs do (grey bar), whereas inverted variants show gene expression under red light (red bar).

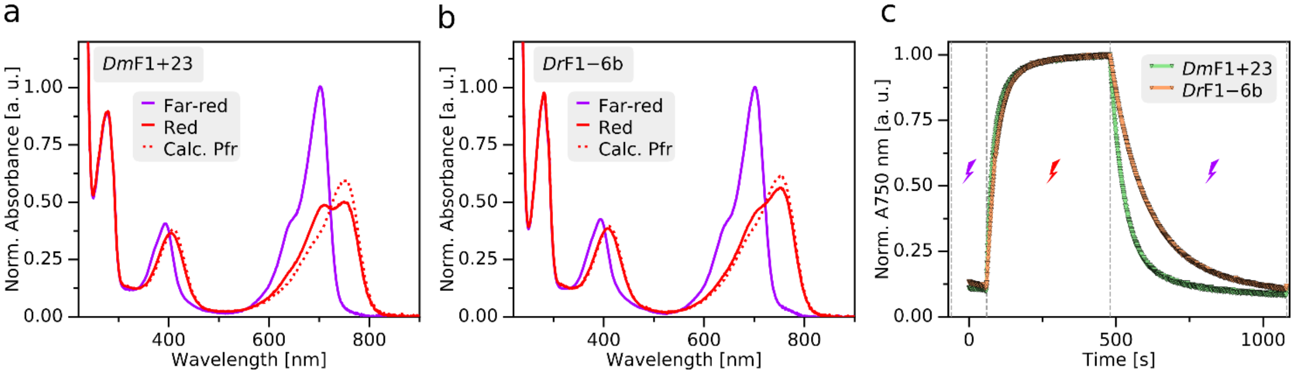

Supplementary Figure 8

Spectroscopic characterization of *Dm*F1+23 and *Dr*F1-6b. **a** UV-vis absorbance spectra of *Dm*F1+23 upon illumination with far-red light (800 nm, purple line) or red light (650 nm, red line). The dotted line denotes the pure Pfr spectrum calculated according to [7]. All spectra were normalized to the absorbance at 700 nm after far-red light exposure. **b** As panel a but for *Dr*F1-6b. **c** The Pr→Pfr photoactivation kinetics of *Dm*F1+23 (green) and *Dr*F1-6b (orange) driven by red light (red flash), and the corresponding Pfr→Pr photoconversion kinetics under far-red light (purple flash). The kinetics were monitored by the absorbance at 750 nm and evaluated according to single-exponential functions. Source data are provided as a Source Data file.

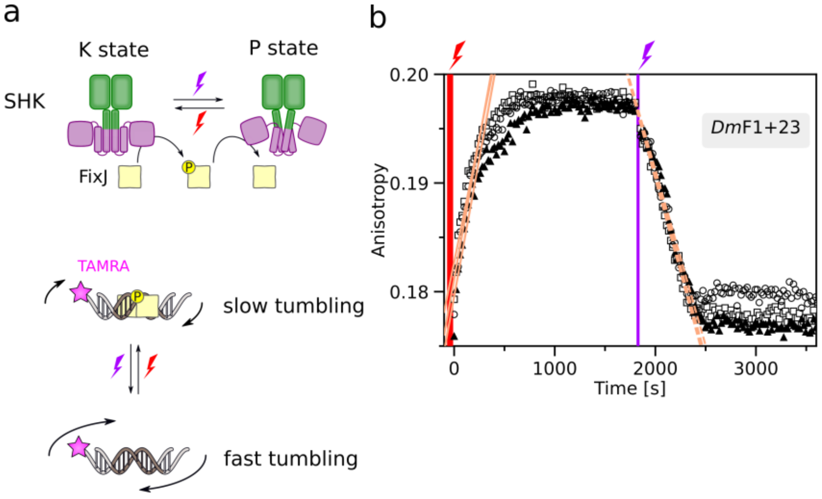

Supplementary Figure 9

Histidine kinase and phosphatase activity of *Dm*F1+23 and *Dr*F1-6b as monitored by fluorescence anisotropy. **a** Schematic of the assay. Red and far-red light interconvert *Dm*F1 and *Dr*F1 between their Pr and Pfr states which, respectively, possess net phosphatase (P state) and net kinase activity (K state) towards the response regulator FixJ. Once phosphorylated, FixJ binds to its cognate operator sequence embedded within a double-stranded DNA fragment labeled with the TAMRA fluorophore. Phospho-FixJ binding decelerates the rotational tumbling of the DNA fragment and thus incurs an increase in TAMRA fluorescence anisotropy. **b** Experimental results for *Dm*F1+23 in triplicates (n = 3, white circles, white squares, black triangles). See Fig. 4d for details. **c** As panel b but for *Dr*F1-6b. Source data are provided as a Source Data file.

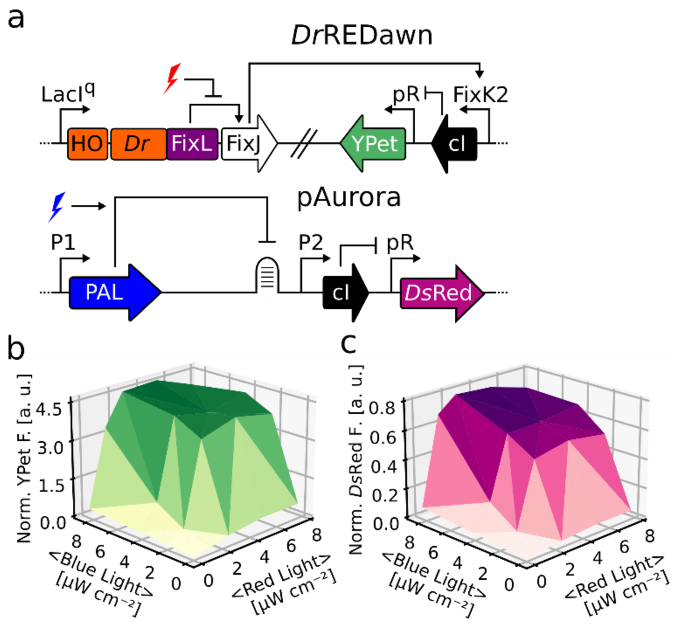

Supplementary Figure 10

Multimodal control of bacterial gene expression by red and blue light. **a** Schematics of the red-light-responsive *Dr*REDawn [1] and the blue-light-responsive pAurora circuits [8]. **b-c** Bacteria harboring both pAurora-*Ds*Red and *Dr*REDawn-YPet were incubated under different red and blue light intensities. The YPet (panel b, green) and *Ds*Red (panel c, pink) fluorescence readings were normalized to the optical density of the bacterial cultures at 600 nm (OD_{600}) and corrected for background fluorescence. Data represent the mean of n = 3 biologically independent replicates. Light intensities are averaged over the duty cycle as marked by angled brackets. Source data are provided as a Source Data file.

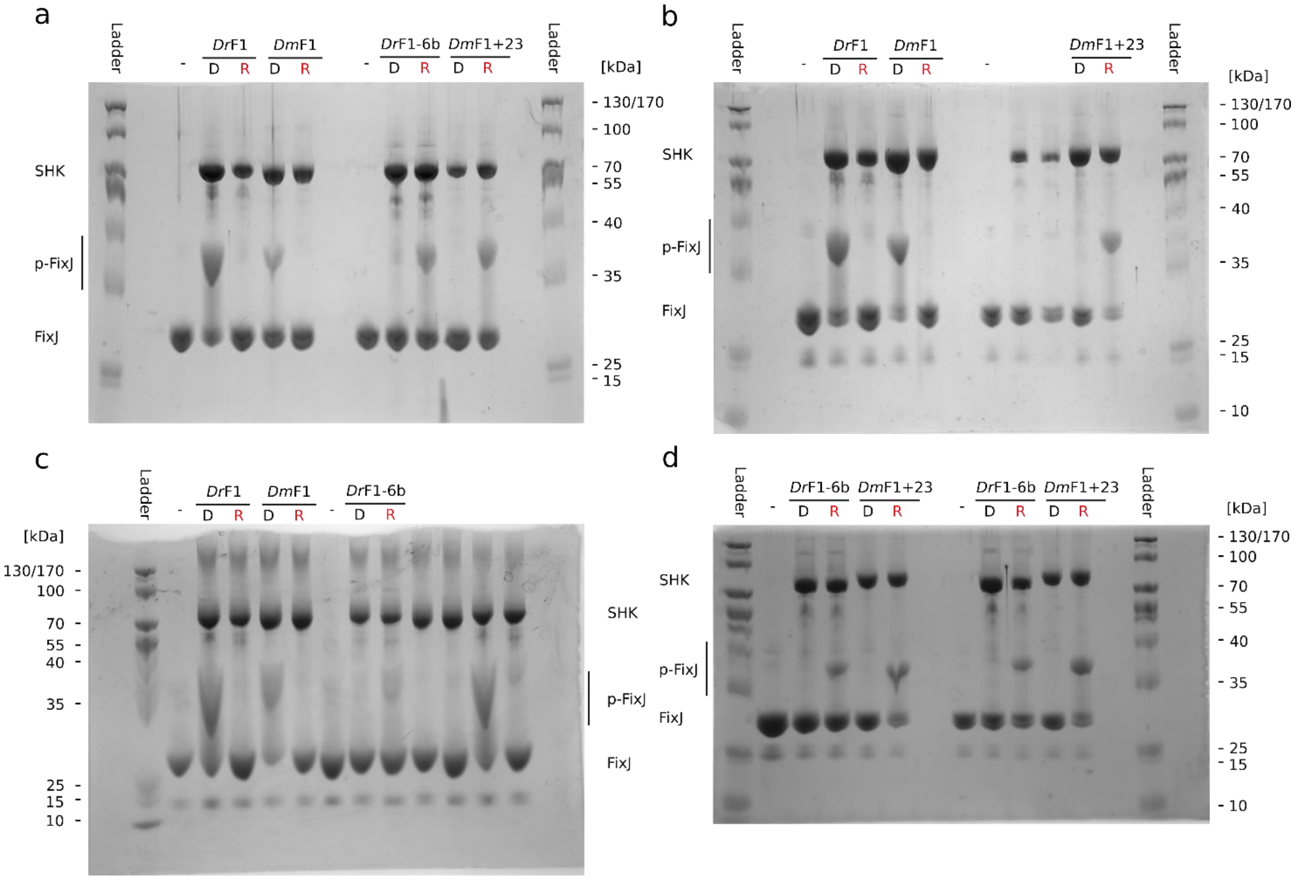

Supplementary Figure 11

a Uncropped version of the Phos-tag gels shown in Fig. 2e and 4c. The leftmost and rightmost lanes contain a marker with the apparent molecular weights indicated. **b-d** Replicate experiments. Unlabeled lanes in panel c contain samples unrelated to the present study.

Supplementary References

1. Multamäki, E. *et al.* Optogenetic Control of Bacterial Expression by Red Light. *ACS Synth. Biol.* **11**, 3354–3367 (2022).
2. Wahlgren, W. Y. *et al.* Structural mechanism of signal transduction in a phytochrome histidine kinase. *Nat Commun* **13**, 7673 (2022).
3. Marina, A., Waldburger, C. D. & Hendrickson, W. A. Structure of the entire cytoplasmic portion of a sensor histidine-kinase protein. *Embo J* **24**, 4247–4259 (2005).
4. Sievers, F. *et al.* Fast, scalable generation of high-quality protein multiple sequence alignments using Clustal Omega. *Molecular Systems Biology* **7**, 539 (2011).
5. Finn, R. D. *et al.* Pfam: clans, web tools and services. *Nucleic Acids Res* **34**, D247–D251 (2006).
6. Ohlendorf, R., Schumacher, C. H., Richter, F. & Möglich, A. Library-Aided Probing of Linker Determinants in Hybrid Photoreceptors. *ACS Synth. Biol.* **5**, 1117–1126 (2016).
7. Butler, W. L., Hendricks, S. B. & Siegelman, H. W. ACTTON SPECTRA OF PHYTOCHROME IN VITRO. *Photochemistry and Photobiology* **3**, 521–528 (1964).
8. Ranzani, A. T. *et al.* Light-Dependent Control of Bacterial Expression at the mRNA Level. *ACS Synth. Biol.* **11**, 3482–3492 (2022).
