# Supplementary material for: Leveraging the histidine kinase-phosphatase duality to sculpt two-component signaling
Source: Nat Commun. 2024 Jun 10;15:4876. doi: 10.1038/s41467-024-49251-8 (PMC11164954; doi:10.1038/s41467-024-49251-8)
Supplement: Supplementary file 3 — Reporting summary [file 41467_2024_49251_MOESM3_ESM.pdf]

Corresponding author(s): Möglich, Andreas

Last updated by author(s): 05/11/2024

Reporting Summary

Nature Portfolio wishes to improve the reproducibility of the work that we publish. This form provides structure for consistency and transparency in reporting. For further information on Nature Portfolio policies, see our Editorial Policies and the Editorial Policy Checklist.

Statistics

For all statistical analyses, confirm that the following items are present in the figure legend, table legend, main text, or Methods section.

n/a	Confirmed	
☐	☒	The exact sample size (*n*) for each experimental group/condition, given as a discrete number and unit of measurement
☐	☒	A statement on whether measurements were taken from distinct samples or whether the same sample was measured repeatedly
☒	☐	The statistical test(s) used AND whether they are one- or two-sided<br>*Only common tests should be described solely by name; describe more complex techniques in the Methods section.*
☒	☐	A description of all covariates tested
☒	☐	A description of any assumptions or corrections, such as tests of normality and adjustment for multiple comparisons
☐	☒	A full description of the statistical parameters including central tendency (e.g. means) or other basic estimates (e.g. regression coefficient) AND variation (e.g. standard deviation) or associated estimates of uncertainty (e.g. confidence intervals)
☒	☐	For null hypothesis testing, the test statistic (e.g. *F*, *t*, *r*) with confidence intervals, effect sizes, degrees of freedom and *P* value noted<br>*Give P values as exact values whenever suitable.*
☒	☐	For Bayesian analysis, information on the choice of priors and Markov chain Monte Carlo settings
☒	☐	For hierarchical and complex designs, identification of the appropriate level for tests and full reporting of outcomes
☒	☐	Estimates of effect sizes (e.g. Cohen's *d*, Pearson's *r*), indicating how they were calculated

Our web collection on statistics for biologists contains articles on many of the points above.

Software and code

Policy information about availability of computer code

Data collection	Tecan i-Control 2.0, Bio-Rad ProSort 1.2, BMG Labtech CLARIOstar 5.21 R2
Data analysis	Microsoft Excel 2401, Fit-o-mat 0.948 (can be obtained from https://github.com/TheAngulion/fit-o-mat)<br><br>Python scripts:<br>1. Analysis of linker properties in multiple sequence alignment: https://github.com/TheAngulion/eval_msa<br>2. Evaluation and presentation of two-color reporter-gene experiments: https://github.com/TheAngulion/multiplex<br>3. Primer design for PATCHY library generation: https://github.com/vrylr/PATCHY

For manuscripts utilizing custom algorithms or software that are central to the research but not yet described in published literature, software must be made available to editors and reviewers. We strongly encourage code deposition in a community repository (e.g. GitHub). See the Nature Portfolio guidelines for submitting code & software for further information.

Data

Policy information about availability of data

All manuscripts must include a data availability statement. This statement should provide the following information, where applicable:

- Accession codes, unique identifiers, or web links for publicly available datasets
- A description of any restrictions on data availability
- For clinical datasets or third party data, please ensure that the statement adheres to our policy

Source data are available from https://doi.org/10.5281/zenodo.10680511
Plasmids have been deposited with Addgene under the accession numbers 213701, 213702, and 213703, and are pending release upon publication.

Research involving human participants, their data, or biological material

Policy information about studies with human participants or human data. See also policy information about sex, gender (identity/presentation), and sexual orientation and race, ethnicity and racism.

Reporting on sex and gender	Not applicable.
Reporting on race, ethnicity, or other socially relevant groupings	Not applicable.
Population characteristics	Not applicable.
Recruitment	Not applicable.
Ethics oversight	Not applicable.

Note that full information on the approval of the study protocol must also be provided in the manuscript.

Field-specific reporting

Please select the one below that is the best fit for your research. If you are not sure, read the appropriate sections before making your selection.

☒ Life sciences ☐ Behavioural & social sciences ☐ Ecological, evolutionary & environmental sciences

For a reference copy of the document with all sections, see nature.com/documents/nr-reporting-summary-flat.pdf

Life sciences study design

All studies must disclose on these points even when the disclosure is negative.

Sample size	For all measurements, at least three biological replicates (i.e., independent samples) were analyzed and averaged. No statistical tests were used to determine sample size. The sample sizes are stated for each experiment. They were determined based on the size and distribution of the measurable differences in the experimental observable.
Data exclusions	No data were excluded.
Replication	All experiments were repeated at least three times with similar outcome. Details are provided in the figure legends.
Randomization	Randomization was not applied as the study did not involve group comparison.
Blinding	Blinding was not applied as the study did not involve group comparison.

Reporting for specific materials, systems and methods

We require information from authors about some types of materials, experimental systems and methods used in many studies. Here, indicate whether each material, system or method listed is relevant to your study. If you are not sure if a list item applies to your research, read the appropriate section before selecting a response.

Materials & experimental systems

n/a	Involved in the study
☒	☐ Antibodies
☒	☐ Eukaryotic cell lines
☒	☐ Palaeontology and archaeology
☒	☐ Animals and other organisms
☒	☐ Clinical data
☒	☐ Dual use research of concern
☒	☐ Plants

Methods

n/a	Involved in the study
☒	☐ ChIP-seq
☐	☒ Flow cytometry
☒	☐ MRI-based neuroimaging

Plants

Seed stocks	Not applicable.
Novel plant genotypes	Not applicable.
Authentication	Not applicable.

Flow Cytometry

Plots

Confirm that:

☒ The axis labels state the marker and fluorochrome used (e.g. CD4-FITC).

☒ The axis scales are clearly visible. Include numbers along axes only for bottom left plot of group (a 'group' is an analysis of identical markers).

☒ All plots are contour plots with outliers or pseudocolor plots.

☐ A numerical value for number of cells or percentage (with statistics) is provided.

Methodology

Sample preparation	Bacterial cultures were grown overnight at 37°C under different illumination conditions. The cultures were then diluted in sheath fluid (i.e., PBS) and directly analyzed by flow cytometry.
Instrument	Bio-Rad S3e
Software	ProSort 1.2
Cell population abundance	Not applicable as the study did not involve cell sorting. Flow cytometry was only applied to characterize the bacterial cell population.
Gating strategy	Not applicable as the study did not involve cell sorting. Flow cytometry was only applied to characterize the bacterial cell population.

☐ Tick this box to confirm that a figure exemplifying the gating strategy is provided in the Supplementary Information.
